# Effect of copper ion and soil humic acid on biodegradation of decabromodiphenyl ether (BDE‐209) by *Pseudomonas aeruginosa*

**DOI:** 10.1002/mbo3.439

**Published:** 2017-01-19

**Authors:** Yu Liu, Aijun Gong, Lina Qiu, Jingrui Li, Fukai Li

**Affiliations:** ^1^ School of Chemistry and Biological EngineeringUniversity of Science and Technology BeijingBeijingChina; ^2^ Institute of BiotechnologyDaqing Branch of Heilongjiang Academy of ScienceDaqingChina; ^3^ Beijing Key Laboratory for Science and Application of Functional Molecular and Crystalline MaterialsUniversity of Science and Technology BeijingBeijingChina

**Keywords:** BDE‐209, biodegradation, copper ion, e‐waste‐contaminated soil, *Pseudomonas aeruginosa*, soil humic acid

## Abstract

*Pseudomonas aeruginosa* is a good environmental microorganism capable of degrading decabromodiphenyl ether (BDE‐209). This paper studied the effect of Cu^2+^ and humic acid (HA) extracted from e‐waste contaminated soils on biodegradation of BDE‐209 by *P. aeruginosa*. The adsorption isotherms of Cu^2+^ on HA, the crude enzyme activity, cell surface morphology, and biodegradation pathway were also investigated. The results showed that BDE‐209 biodegradation by *P. aeruginosa* was inhibited at Cu^2+^ concentrations above 5 mg L^−1^, but exhibited the best effect at the condition of 40 mg L^−1^ Cu^2+^ + 3 g L^−1^ HA. At the condition of 40 mg L^−1^ Cu^2+^ + 3 g L^−1^ HA, 97.35 ± 2.33% of the initial BDE‐209 was degraded after 5 days, debromination efficiency was 72.14 ± 1.89%, crude enzyme activity reached the maximum of 0.519 ± 0.022U g^−1^ protein, cell surface of *P. aeruginosa* was smooth with normal short‐rod shapes, and biodegradation pathway mainly include debromination, hydroxylation, and cleavage of the diphenyl ether bond. It was suggested that soil HA could eliminate the toxic effect of high Cu^2+^ concentrations and biodegradation of BDE‐209 was improved by synergistic effect of HA and Cu^2+^.

## 1 INTRODUCTION

Electronic waste (e‐waste) is becoming one of the fastest growing components of municipal solid waste in today's world (Kim et al., 2015). It often contains significant quantities of heavy metals (eg, Cu and Pb), reusable precious metals (eg, Au and Ag), and nonmetallic components consisting of brominated flame retardants, resins, ceramics, and plastics (Natarajan, Ting, & Routti, 2015). Over the past 20 years, recycling of e‐waste (especially informal sector recycling) has caused serious environmental problems and received enormous attention globally, especially in developing countries (Chi, Wang, & Reuter, 2014; Fu et al., 2008; Song, Li, & Rout, 2015).

China is the most affected country by informal e‐waste recycling (Zeng, Xu, Boezen, & Huo, 2016). Research in China has shown that the informal e‐waste recycling processes release significant amounts of polybrominated diphenyl ethers (PBDEs), in which decabromodiphenyl ether (BDE‐209) is generally predominant (Chen et al., 2016). For instance, Nie et al. investigated the levels of 21 kinds of PBDEs from soil samples surrounding the e‐waste burning site in Qingyuan, China. Average concentrations of PBDEs were 2,283 ng g^−1^ dw, with values ranging from 5.27 to 22,110 ng g^−1^ dw, while the concentrations of BDE‐209 ranged from 4.40 to 21,467 ng g^−1^ dw with an average of 2,162 ng g^−1^ dw (Nie et al., 2015). BDE‐209, toxic to microorganisms, animals, and humans, is a persistent compound and has accumulated in a diversity of environmental matrices. So far, BDE‐209 can be degraded by different methods, such as reductive degradation, oxidative degradation, and ultraviolet photolysis.

Microbial biodegradation has the advantage of being low cost and environment friendly. *Pseudomonas aeruginosa*, capable of degrading BDE‐209, has great potential for bioremediation application in e‐waste‐contaminated soils. For example, Shi et al. used *P. aeruginosa* to degrade BDE‐209 and the results showed excellent effect (Shi et al., 2013). But, several studies have reported that the presence of Cu^2+^ has influence on BDE‐209 biodegradation. BDE‐209 degradation is stimulated at low concentrations of Cu^2+^, whereas inhibited at higher levels of Cu^2+^. For instance, Xu et al. reported that white‐rot fungus *Phlebia lindtneri* could degrade 77.3% of BDE‐209 within 30 days. Degradation was stimulated at low concentrations of Cu^2+^ (≤5.0 mg L^−1^) and inhibited at higher concentrations (Xu, Wang, & Letcher, 2014). In China, Cu^2+^ is also found in high concentrations in e‐waste‐contaminated soils, such as Guiyu (Cu^2+^: 787.7 mg kg^−1^), Taizhou (Cu^2+^: 158.1 mg kg^−1^) (Song, Li, & Hu, 2014). So, if the strain *P. aeruginosa* is applied, its ability to degrade BDE‐209 is most likely to be inhibited. Humic acid (HA) is ubiquitous in the soil as the critical component of natural organic matter (Vidali, Remoundaki, & Tsezos, 2010). With abundant polar functional groups (eg, –COOH and –OH), soil HA exhibits intensive adsorption ability toward heavy metal ions (Sounthararajah, Loganathan, Kandasamy, & Vigneswaran, 2015). Up to now, the effect of copper ion and soil HA on biodegradation of BDE‐209 by *P. aeruginosa* has never been reported.

The main objective of the present work was to study the effect of Cu^2+^ and HA extracted from e‐waste‐contaminated soils on biodegradation of BDE‐209 by *P. aeruginosa*, principally focusing on the degradation efficiency, debromination efficiency, crude enzyme activity, and possible biodegradation pathway. The sorption capacity of Cu^2+^ on HA was also investigated.

## 2 MATERIALS AND METHODS

### 2.1 Chemicals

Cupric nitrate (Cu(NO_3_)_2_·3H_2_O, AR) was provided by Tianjing Kemiou Chemical Reagent Co., Ltd (China). HA was extracted from e‐waste‐contaminated soils in China. BDE‐209 with a purity of >99% was provided from Alfa Aesar (China). Standard of BDE‐209 was obtained from Sigma (USA). Standards of ^13^C‐BDE‐209 and ^13^C‐PCB‐141 were purchased from Wellington Labs (Canada). Osmium tetroxide was purchased from Beijing Best Technology co., Ltd (China). All other reagents (AR) were purchased from Sinopharm Chemical Reagent Beijing Co., Ltd (China).

### 2.2 Strain and cultivation conditions

Sludge samples were collected from PBDEs‐contaminated sites in China. Enrichment was carried out by cultivating the samples in mineral salt medium (MSM) (Tang et al., 2016) containing 10 mg L^−1^ BDE‐209 on a rotary shaker at 200 rpm and 30°C for 5 days. A total quantity of 2 ml of enrichment culture was orderly transferred to fresh MSM containing 10, 20, 50, 100 mg L^−1^ BDE‐209, respectively. After 5 days, enrichment was plated onto solid medium to obtain single colonies. Finally, a BDE‐209‐degrading strain was isolated and showed high capacity. The strain was identified as *P. aeruginosa* based on morphological, cultural, physiological characteristics, and 16S rDNA sequence analysis.

*Pseudomonas aeruginosa* were cultivated in a defined mineral salt medium (DMSM) without addition of Cu^2+^, pH value of 7.0, with the following composition (all in g L^−1^) : 5.3 KH_2_PO_4_, 10.6 K_2_HPO_4_, 10.0 KNO_3_, 2.0 Na_2_SO_4_, 0.18 MgSO_4_, 0.086 CaCl_2_, and trace elements solution 1 ml (containing (g L^−1^): 5.74 ZnSO_4_·7H_2_O, 3.96 MnCl_2_·4H_2_O, 1.24 H_3_BO_3_, 0.85 Co(NO_3_)_2_, 0.83 NH_4_MoO_4_, and 0.22 FeSO_4_·7H_2_O). BDE‐209 (20 mg L^−^1) was used as the sole metabolic carbon source in all tests, and cultivation temperature was 35°C. Cell density was described using absorbance at 600 nm (OD_600_), which were determined by an ultraviolet–visible spectrophotometer (UV‐2550, Shimadzu Co. Ltd, Japan).

### 2.3 Sorption of Cu^2+^ on HA

The adsorption isotherms of Cu^2+^ on HA were conducted using batch experiments at equilibrium pH of 7.0. The equilibrium pH in solution was adjusted using HNO_3_ or NaOH. Ten milligram of HA and 25 ml Cu(NO_3_)_2_ solutions with initial Cu^2+^ concentrations of 1–200 mg L^−1^ were mixed with 200 mg NaNO_3_ in 50 ml polyethylene centrifuge tubes. The ionic strength of the solution was controlled by adding NaNO_3_. The mixtures were shaken in a thermostat shaker at 150 rpm and 25 ± 1°C for 24 hr. The suspensions were separated by centrifugation at 5,000 rpm for 20 min, and then filtered through 0.45‐μm filters. Finally, the Cu^2+^ concentrations in the supernatants were determined by an acetylene‐air flame atomic absorption spectrophotometer (AA‐6800, Shimadzu Co. Ltd, Japan). The amounts of adsorbed Cu^2+^ were calculated by the mass balance Equation (1) (Huang et al., 2014). The adsorption isotherm data were fitted with the Langmuir model and Freundlich model. The Langmuir adsorption isotherm was expressed as the Equation (2), and the Freundlich adsorption isotherm was explained by the Equation (3) (Khalili, Al‐Banna, & Yu, 2015):

(1) $$q_{e}=\frac{\left(C_{0}-C_{e}\right)V}{m}$$

(2) $$q_{e}=\frac{q_{m}k_{L}C_{e}}{1+k_{L}C_{e}}$$

(3) $$q_{e}=k_{F}C_{e}^{1/n}$$

where *q*_e_ (mg g^−1^) is the amount of Cu^2+^ adsorbed at equilibrium, *C*_0_ and *C*_e_ (mg L^−1^) are the initial and the equilibrium Cu^2+^ concentrations, *V* (L) is the volume of the solution, *m* (g) was the mass of the adsorbent, *q*_m_ (mg g^−1^) and *k*_L_ (L mg^−1^) are Langmuir constants related to the maximum adsorption capacity and energy or intensity of adsorption, respectively, *k*_F_ (mg g^−1^ (mg L^−1^)^−1/*n*^) and 1/*n* are the Freundlich constants representing the adsorption capacity and the adsorption intensity or heterogeneity of adsorbent, respectively.

### 2.4 Biodegradation system of BDE‐209 by *P. aeruginosa*

Batch biodegradation tests were performed in triplicate in 250 ml Erlenmeyer flasks. In each case, 150 ml DMSM and 3.0 mg BDE‐209 (20 mg L^−1^) were placed in a flask and mixed ultrasonically for 10 min to get a suspension. Then, 60 mg live *P. aeruginosa* cells were added to the suspension in a super clean bench and incubated at 35°C on a rotary shaker at 200 rpm for 5 days. Dead *P. aeruginosa* cells were substituted for live cells in the system served as control.

### 2.5 Effect of Cu^2+^ and HA on the growth of *P. aeruginosa*

Batch experiments were conducted by adding 0–1 ml Cu(NO_3_)_2_ solutions (0.2 mol L^−1^) and 450, 900 mg HA into the biodegradation system of BDE‐209, and the final concentrations of Cu^2+^ and HA were set as follows: 0–80 mg L^−1^ Cu^2+^ + 3 g L^−1^ HA, 0–80 mg L^−1^ Cu^2+^ + 6 g L^−1^ HA. In order to assess the effect of Cu^2+^ and HA on the growth of *P. aeruginosa*, OD_600_ values were observed at 0–80 mg L^−1^ Cu^2+^, 0–80 mg L^−1^ Cu^2+^ + 3 g L^−1^ HA, 0–80 mg L^−1^ Cu^2+^ + 6 g L^−1^ HA in 5 days.

### 2.6 Effect of Cu^2+^ and HA on biodegradation of BDE‐209 by *P. aeruginosa*

To investigate the influence of Cu^2+^ and HA on BDE‐209 biodegradation by *P. aeruginosa*, degradation efficiency and debromination efficiency were determined at 0–80 mg L^−1^ Cu^2+^, 0–80 mg L^−1^ Cu^2+^ + 3 g L^−1^ HA, 0–80 mg L^−1^ Cu^2+^ + 6 g L^−1^ HA after 5 days. Degradation efficiency of BDE‐209 and debromination efficiency were calculated according to the following equations:

(4) $$C=\frac{C_{C}-C_{S}}{C_{C}}\times 100\%$$

(5) $$M=\frac{M_{\mathrm{Br}}}{M_{T}}\times 100\%$$

where *C* is the degradation efficiency of BDE‐209, *C*_C_ the initial BDE‐209 concentration, *C*_S_ the BDE‐209 concentration in biodegradation test, *M* debromination efficiency of BDE‐209, *M*_Br_ the concentration of bromide ions released, *M*_T_ the theoretical bromide ion concentration from the complete debromination of the substrate.

### 2.7 Extraction of crude enzyme

One gram *P. aeruginosa* cells were placed in a 50 ml centrifuge tube, suspended in the ice‐cold 12 ml NaH_2_PO_4_–Na_2_HPO_4_ buffer (*c* = 0.05 mol L^−1^, pH = 9.0), were subjected to 100 rounds of sonication in an ice water bath for 3 s followed by cooling for 6 s (the time of 10 rounds was 1 min). The debris was removed by centrifugation at 11,400 g for 10 min. The supernatant was filtered with 0.22 μm pore‐size filters and the filtrate was crude enzyme. The protein was measured by Bradford method at 595 nm by UV–Vis spectrophotometer using bovine serum albumin as a standard (Bradford, 1976).

### 2.8 Biodegradation system of BDE‐209 by crude enzyme

Batch biodegradation tests were performed in triplicate in 250 ml Erlenmeyer flasks. In each case, 150 ml NaH_2_PO_4_–Na_2_HPO_4_ buffer (*c* = 0.05 mol L^−1^, pH = 9.0) and 3.0 mg BDE‐209 (20 mg L^−1^) were placed in a flask and mixed ultrasonically for 10 min to get a suspension. Then, 100 mg crude enzyme was added to the suspension in a super clean bench and incubated at 35°C on a rotary shaker at 200 rpm for 1 hr.

### 2.9 Effect of Cu^2+^ and HA on the crude enzyme activity of *P. aeruginosa*

In order to investigate the influence of Cu^2+^ and HA on the crude enzyme activity of *P. aeruginosa*, Cu(NO_3_)_2_ and HA at various concentrations as described above were added into the biodegradation system of BDE‐209 by *P. aeruginosa*, so the initial concentrations of Cu^2+^ and HA were set as follows: 0–80 mg L^−1^ Cu^2+^ + 3 g L^−1^ HA, 0–80 mg L^−1^ Cu^2+^ + 6 g L^−1^ HA. After 24 hr, *P. aeruginosa* cells were harvested to extract crude enzyme and the crude enzyme activity was assayed. One unit (U) of crude enzyme activity was defined as the amount of the enzyme catalyzing the degradation of 1 μmol BDE‐209 per min at 35°C. The crude enzyme activity was calculated as U g^−1^ protein.

### 2.10 Scanning electron microscope (SEM) observation

Surface morphology of *P. aeruginosa* was observed under a ZEISS‐EVO18 scanning electron microscope (Carl Zeiss NTS, Germany), the cells in the biodegradation system containing 0–80 mg L^−1^ Cu^2+^, 0–80 mg L^−1^ Cu^2+^ + 3 g L^−1^ HA, 0–80 mg L^−1^ Cu^2+^ + 6 g L^−1^ HA were prepared and fixed with 2.5% glutaraldehyde in phosphate‐buffered solution (PBS) for 4 hr, then washed separately four times with PBS, postfixed with 1% osmium tetroxide for 1 hr, and rinsed again with deionized water, dehydrated using a gradient series of water‐ethanol solutions (30%, 50%, 70%, 80%, 90%, and 100%) two times for 60 min each. Finally, the specimens were mounted on metal stubs and stored in a desiccator for 36 hr, and sputter‐coated with a gold layer. The SEM observation was done under the following conditions: Extra high tension (EHT) = 20.00 kV, Working distance (WD) = 14.0 mm, Signal A = SE1.

### 2.11 Extraction and analytical methods

At each sampling time, all samples were extracted (1:1, *v*/*v*) with toluene in a separating funnel by vigorously shaking for 15 min and allowed to set until phase separation. A surrogate standard (^13^C‐BDE‐209) for recovery estimation was spiked prior to extraction. Then, the organic phase was collected and dried with anhydrous sodium sulfate. The aqueous phase was extracted again as described above. The combined organic phases were concentrated under reduced pressure at 40°C. After being evaporated to approximately 5 ml, ^13^C‐PCB‐141 was added as the internal standard before gas chromatography–mass spectrometer (GC–MS) analyses.

The quantification of BDE‐209 and the lower brominated PBDEs were analyzed using 7890–5975c GC–MS (Agilent, USA) equipped with a DB‐5 MS column (60 m × 0.25 mm × 0.25 μm). Helium was used as the carrier gas at a flow rate of 1 ml min^−1^. The injection volume was 1 μl. The column temperature program started at 60°C (held for 1 min), then increased 20°C min^−1^ to 220°C (held for 1 min), increased 5°C min^−1^ to 250°C (held for 1 min), increased 20°C min^−1^ to 280°C (held for 10 min), and finally increased 15°C min^−1^ to 300°C (held for 10 min). Mass spectrometer conditions were as follows: electron impact ionization, ionization energy 70 eV, full scan.

The concentration of bromide ion was measured via ICS‐5000 ion chromatography (Dionex, China) using a Dionex Ionpac AS11‐HC analytical column (4 × 250 mm). The mobile phase was 30% NaOH at a flow rate of 1.2 ml min^−1^. The column temperature was maintained at 30°C, and the sample injection volume was 20 μl.

### 2.12 Quality assurance and quality control

A procedural blank and a duplicated sample were run with each batch of 10 samples to assess potential sample contamination and the repeatability of the analysis. The surrogate recoveries for ^13^C‐BDE‐209 ranged from 92.3% to 118.7% (RSD <,  15%, RSD: relative standard deviation). The limit of detection (LOD) was defined as three times the concentration of analyte in the sample producing a peak with the ratio of signal to noise of 3 (peak‐to‐peak). For the target analytes (BDE‐209), which were not detected in procedural blanks, LOD for BDE‐209 was 50 ng g^−1^.

### 2.13 Statistical analysis

In this study, all experiments were performed in triplicate flasks to get reliable data, and the results presented were the mean values of the three replicates. The standard deviations for measurements ranged from 1.0% to 5.0%.

## 3 RESULTS

### 3.1 Adsorption isotherms of Cu^2+^ on HA

**Figure 1:**
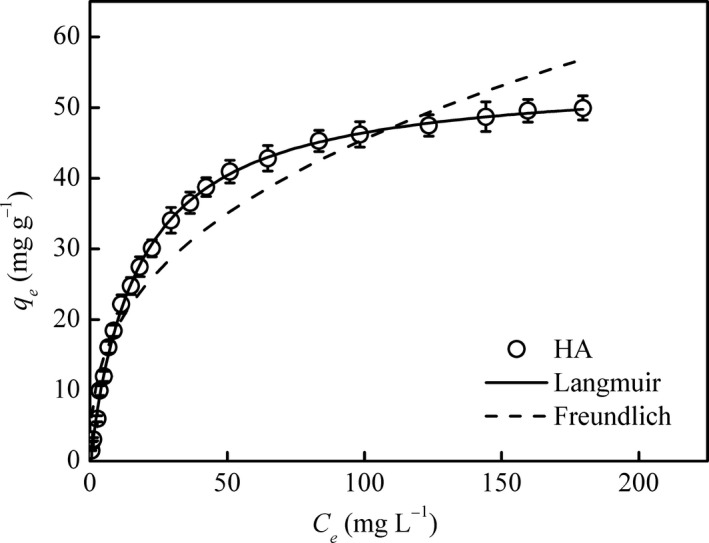
Langmuir and Freundlich models fitted isotherms of Cu^2+^ on humic acid (HA) at pH 7.0

The adsorption isotherms of Cu^2+^ on HA at pH 7.0 are presented in Figure 1, and the adsorption isotherm parameters of the Langmuir and Freundlich equations obtained by fitting the isotherms are listed in Table 1. HA displayed similar shapes of the adsorption isotherms. The amount of adsorbed Cu^2+^ on HA increased with the increase in Cu^2+^ concentrations. As listed in Table 1, *R* ^2^ of the Langmuir and Freundlich equations were 0.999 and 0.939, respectively. The large *R* ^2^ indicated that Langmuir model had the best fit for the adsorption isotherm of Cu^2+^ on HA. Significant deviations from experimental data were observed for Freundlich model. Based on the Langmuir equation, the maximum adsorption quantity of Cu^2+^ (*q*_m_) on HA was 54.6 ± 0.4, and the Langmuir constants (*k*_L_) was 0.0570 ± 0.0012.

**Table 1:** Adsorption isotherm parameters derived from Langmuir and Freundlich equations for Cu^2+^ on humic acid

	Langmuir equation^a^			Freundlich equation^b^		
	*q*_m_	*k*_L_	*R* ^2^	*k*_F_	1/*n*	*R* ^2^
HA	54.6 ± 0.4	0.0570 ± 0.0012	0.999	7.94 ± 0.94	0.379 ± 0.028	0.939
All estimated parameter values and their standard errors were determined by commercial software (Origin 8.5)						

a *q*_m_, maximum adsorption quantity (mg g^−1^); *k*_L_, Langmuir constants (L mg^−1^); *R* ^2^, coefficient of determination.

b *n*, Freundlich constants (dimensionless); *k*_F_, Freundlich constants (mg g^−1^ (mg L^−1^)^−1/*n*^).

### 3.2 Effect of Cu^2+^ and HA on the growth of *P. aeruginosa*

**Figure 2:**
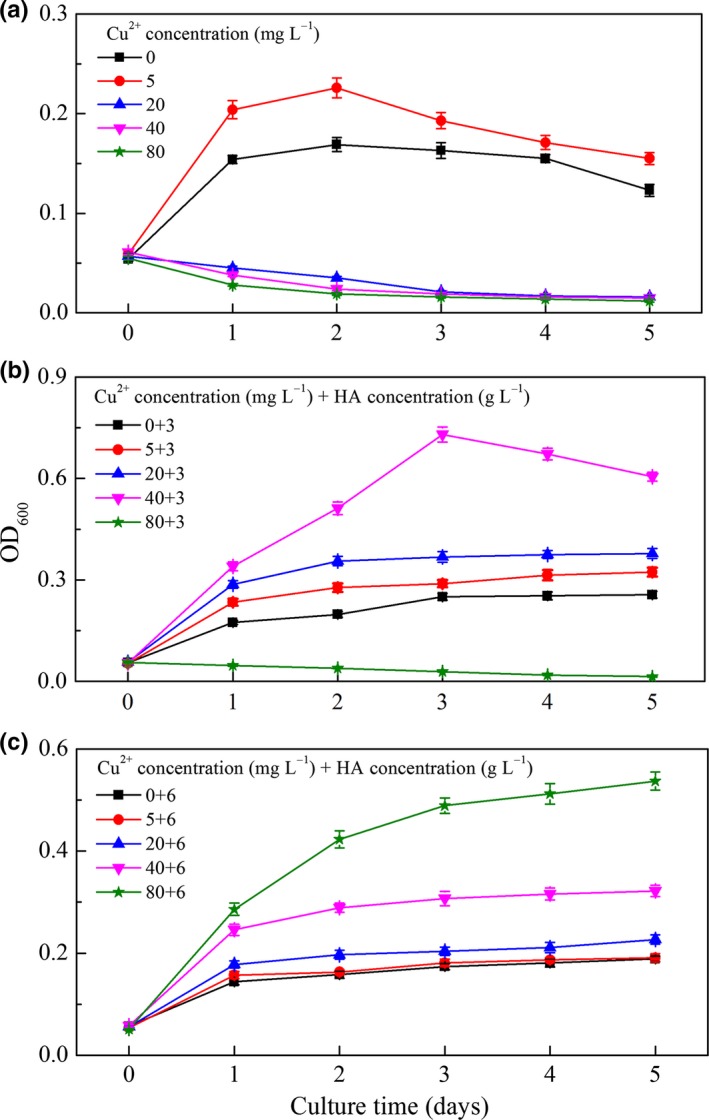
Impact of Cu^2+^ and humic acid on the growth of *Pseudomonas aeruginosa* in 5 days. (a) 0–80 mg L^−1^ Cu^2+^; (b) 0–80 mg L^−1^ Cu^2+^ + 3 g L^−1^ humic acid (HA); (c) 0–80 mg L^−1^ Cu^2+^ + 6 g L^−1^ HA

The effect of Cu^2+^ and HA on the growth of *P. aeruginosa* is shown in Figure 2a–c. It can be seen from Figure 2a that *P. aeruginosa* growth was accelerated when Cu^2+^ concentration was 5 mg L^−1^, but inhibited at higher Cu^2+^ concentrations (>5 mg L^−1^). At day 2, *P. aeruginosa* growth at Cu^2+^ concentration of 5 mg L^−1^ reached the highest OD_600_ (0.226 ± 0.010). Figure 2b shows the growth of *P. aeruginosa* in the presence of 3 g L^−1^ HA. *P. aeruginosa* growth was accelerated at 5, 20, and 40 mg L^−1^ Cu^2+^, but inhibited at 80 mg L^−1^ Cu^2+^. Under the condition of 40 mg L^−1^ Cu^2+^ + 3 g L^−1^ HA, OD_600_ reached the maximum value (0.730 ± 0.022) at day 3. As can be seen from Figure 2c, even though *P. aeruginosa* growth was stimulated at 80 mg L^−1^ Cu^2+^ in the presence of 6 g L^−1^ HA, the OD_600_ were smaller than that at the condition of 40 mg L^−1^ Cu^2+^ + 3 g L^−1^ HA. Collectively, these results suggest that the inhibition effect of high Cu^2+^ concentrations on the growth of *P. aeruginosa* was eliminated by adding appropriate amount of HA, and *P. aeruginosa* had the fastest growth at the condition of 40 mg L^−1^ Cu^2+^ + 3 g L^−1^ HA.

### 3.3 Effect of Cu^2+^ and HA on the degradation of BDE‐209 by *P. aeruginosa*

**Figure 3:**
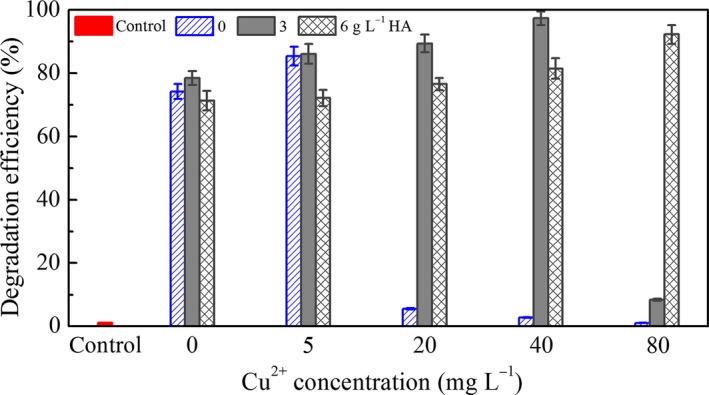
Influence of Cu^2+^ and humic acid (HA) on the degradation of BDE‐209 by *Pseudomonas aeruginosa* for 5 days. Control: 0 mg L^−1^ Cu^2+^, with addition of dead *P. aeruginosa* cells

The effect of Cu^2+^ and HA on the degradation of BDE‐209 by *P. aeruginosa* is presented in Figure 3. In the absence of HA, degradation efficiency was increased when Cu^2+^ concentration was 5 mg L^−1^, but decreased when Cu^2+^ concentration was high (>5 mg L^−1^). Adding HA did not change degradation efficiency too much at low Cu^2+^ concentration. When the concentration of Cu^2+^ was high, degradation efficiency obviously increased with the addition of HA. These results also demonstrated that a certain amount of HA could greatly improve BDE‐209 degradation by *P. aeruginosa* at high Cu^2+^ concentrations. Among all conditions, the concentration of 40 mg L^−1^ Cu^2+^ + 3 g L^−1^ HA exhibited the highest degradation efficiency (97.35 ± 2.33%).

### 3.4 Effect of Cu^2+^ and HA on the debromination of BDE‐209 by *P. aeruginosa*

**Figure 4:**
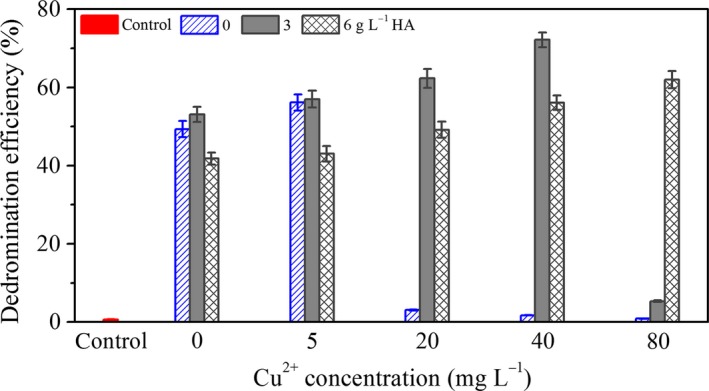
Influence of Cu^2+^ and humic acid (HA) on the debromination of BDE‐209 by *Pseudomonas aeruginosa* for 5 days. Control: 0 mg L^−1^ Cu^2+^, with addition of dead *P. aeruginosa* cells

The effect of Cu^2+^ and HA on the debromination of BDE‐209 is displayed in Figure 4. In the absence of HA, the debromination efficiency of *P. aeruginosa* at 5 mg L^−1^ Cu^2+^ was a bit higher than that at 0 mg L^−1^ Cu^2+^, but decreased at a higher concentration of Cu^2+^. This result also confirmed that the debromination of BDE‐209 by *P. aeruginosa* was stimulated at low concentrations of Cu^2+^ (5 mg L^−1^) and inhibited at higher concentrations of Cu^2+^ (>5 mg L^−1^). By adding 3 g L^−1^ HA, debromination efficiency was improved and reached the maximum of 72.14 ± 1.89% at 40 mg L^−1^ Cu^2+^. No inhibitory effect on debromination was observed after adding 6 g L^−1^ HA. This also indicated that a certain amount of HA could greatly improve the BDE‐209 debromination of *P. aeruginosa* at high Cu^2+^ concentrations (>5 mg L^−1^).

### 3.5 Effect of Cu^2+^ and HA on the crude enzyme activity of *P. aeruginosa*

**Figure 5:**
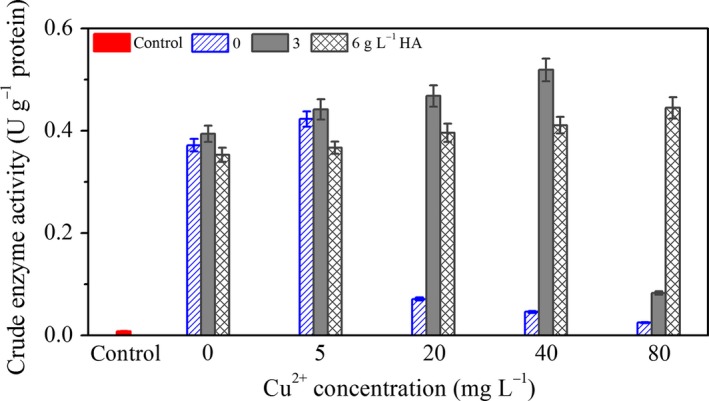
Effect of Cu^2+^ and HA on the crude enzyme activity of *Pseudomonas aeruginosa* for 24 hr. Control: 0 mg L^−1^ Cu^2+^, with addition of dead *P. aeruginosa* cells

It is well known that crude enzyme is intracellular enzyme mixtures, and it contains BDE‐209 degradation enzyme which plays a key role in biodegradation by *P. aeruginosa* (Shi et al., 2013). So far, we cannot directly determine the activity of BDE‐209 degradation enzyme, because we do not know which enzyme has the ability of BDE‐209 degradation in crude enzyme of *P. aeruginosa*. Because crude enzyme activity reflects the ability of BDE‐209 biodegradation by *P. aeruginosa*, we studied crude enzyme activity instead of BDE‐209 degradation enzyme in this work. As shown in Figure 5, effect of Cu^2+^ and HA on the crude enzyme activity is consistent with the results obtained above. The maximum value of crude enzyme activity was 0.519 ± 0.022 U g^−1^ protein under the condition of 40 mg L^−1^ Cu^2+^+3 g L^−1^ HA.

### 3.6 Effect of Cu^2+^ and HA on the surface morphology of *P. aeruginosa*

**Figure 6:**
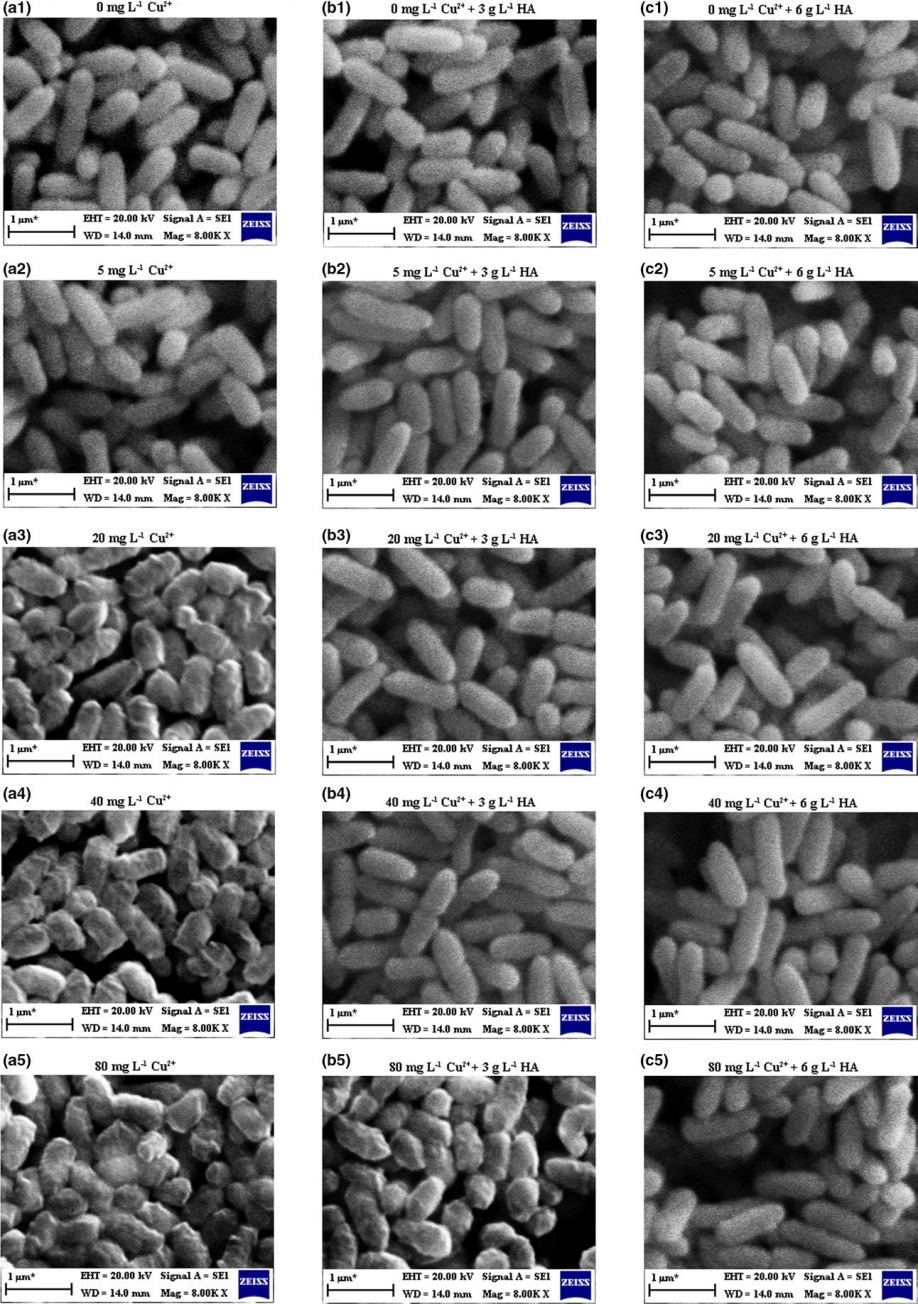
Scanning electron microscope micrographs of *Pseudomonas aeruginosa* at concentrations of 0–80 mg L^−1^ Cu^2+^, 0–80 mg L^−1^ Cu^2+^ + 3 g L^−1^ humic acid (HA), and 0–80 mg L^−1^ Cu^2+^ + 6 g L^−1^ HA. (a‐1–5): 0, 5, 20, 40, 80 mg L^−1^ Cu^2+^; (b‐1–5): 0, 5, 20, 40, 80 mg L^−1^ Cu^2+^ + 3 g L^−1^ HA; (c‐1–5): 0, 5, 20, 40, 80 mg L^−1^ Cu^2+^ + 6 g L^−1^ HA

As mentioned above, the growth of *P. aeruginosa* and the biodegradation of BDE‐209 were obviously stimulated at Cu^2+^ concentrations of 5 mg L^−1^, but severely inhibited at Cu^2+^ concentrations above 5 mg L^−1^. After adding HA, the growth of *P. aeruginosa* and the biodegradation of BDE‐209 were maximally stimulated at the concentrations of 40 mg L^−1^ Cu^2+^ + 3 g L^−1^ HA. In order to deeply understand the differences of *P. aeruginosa* under the above‐mentioned conditions, cell surface morphology was observed. The SEM micrographs of *P. aeruginosa* under different conditions are presented in Figure 6. Cell surface of *P. aeruginosa* was smooth with short‐rod shapes in a‐1 and a‐2, while rough and irregular in a‐3, a‐4, and a‐5. These deformations were attributed to the toxic effect of high Cu^2+^ concentrations. By comparing a‐3 and a‐4 with b‐3 and b‐4, it was found that cell surface of *P. aeruginosa* became smooth with short‐rod shapes by adding 3 g L^−1^ HA when the concentration of Cu^2+^ was 20 and 40 mg L^−1^. But in Figure 6b‐5, cell surface of *P. aeruginosa* was also in irregular shapes. In Figure 6c‐1–5, all cell surfaces of *P. aeruginosa* were in normal short‐rod shapes. This demonstrated that the toxic effect of high Cu^2+^ concentrations can be eliminated by adding a certain amount of HA.

### 3.7 Biodegradation pathway of BDE‐209 by *P. aeruginosa*

**Figure 7:**
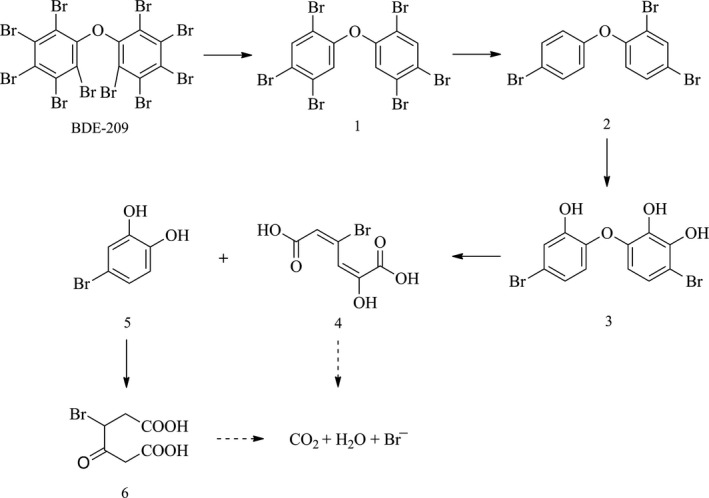
Possible biodegradation pathway of decabromodiphenyl ether (BDE‐209) by *Pseudomonas aeruginosa*

To get a better insight into the fate of BDE‐209, the possible pathways and mechanism of BDE‐209 biodegradation by *P. aeruginosa* in the presence of 40 mg L^−1^ Cu^2+^ + 3 g L^−1^ HA, were proposed based on GC–MS analysis. Lower brominated PBDEs, OH‐PBDEs, and open‐ring products were detected and listed in Table 2. Based on these identified intermediates, one of the possible pathway was proposed in Figure 7. BDE‐209 was initially debrominated to generate BDE‐153 (1), which was further debrominated and underwent hydroxylation reaction to give BDE‐28 (2) and 2,2′,3′‐trihydroxy‐4,4′‐dibromodiphenyl ether (3). Afterward, cleavage and ring‐opening reaction took place, yielding 2‐hydroxy‐4‐bromo‐adipic acid (4), 3‐bromocatechol (5), and 3‐oxo‐4‐bromo‐adipic acid (6). Finally, they were mineralized to produce carbon dioxide and water.

**Table 2:** Chemical structures of decabromodiphenyl ether (BDE‐209) biodegradation products identified by GC–MS analysis in the biodegradation system of *Pseudomonas aeruginosa* after 5 days with initial concentration of 40 mg L^−1^ Cu^2+^ + 3 g L^−1^ humic acid

Number	Biodegradation products	Chemical structure
1	BDE‐153	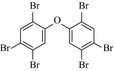
2	BDE‐28	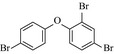
3	2,2′,3′‐Trihydroxy‐4,4′‐dibromodiphenyl ether	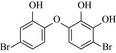
4	2‐Hydroxy‐4‐bromo‐adipic acid	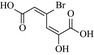
5	3‐Bromocatechol	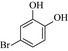
6	3‐oxo‐4‐bromo‐adipic acid	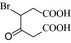

## 4 DISCUSSION

It is well known that Cu^2+^ is an important micronutrient for microorganism growth and metabolism. But, Cu^2+^ has a very narrow optimum concentration range, above which it is toxic to microorganism (Kim, Nevitt, & Thiele, 2008; Tremaroli et al., 2010). Furthermore, Cu^2+^ can obviously influence the microbial degradation of BDE‐209 (Xu et al., 2014). Here, we showed that *P. aeruginosa* growth and the biodegradation of BDE‐209 by *P. aeruginosa* were accelerated when exposed to 5 mg L^−1^ Cu^2+^ but strongly inhibited when exposed to above 5 mg L^−1^ Cu^2+^. This was also confirmed by the variation in the morphology of *P. aeruginosa* (Figure 6a‐1–5). This phenomenon is probably because Cu^2+^ is the cofactor of key enzymes involved in multiple cell activities (Arredondo, Nunez, & Gabrielsen, 2005), so *P. aeruginosa* exhibits good growth and normal short‐rod shapes at low Cu^2+^ concentration. When Cu^2+^ was in excess, Cu^2+^ became active factor of oxidation–reduction reaction, participates in a Fenton reaction, and produces harmful OH^−^. OH^−^ can cause cell membrane lipid peroxidation, DNA/RNA unwinding, finally lead to cell death (Gaetke, 2003). So, at high Cu^2+^ concentration, *P. aeruginosa* cell morphology was rough with irregular shapes, meanwhile *P. aeruginosa* cell growth and the biodegradation of BDE‐209 were strongly inhibited.

Various reactive functional groups in HA are known to absorb contaminant of Cu^2+^ and have a major influence on the fate and transport of toxic Cu^2+^ in environmental systems (El‐Eswed, Khalili, & Raff, 2006). Adsorption of Cu^2+^ on HA is an important subject that attracts long‐lasting attention (Li et al., 2015). However, the effect of Cu^2+^ and soil HA on biodegradation of BDE‐209 by *P. aeruginosa* has never been reported previously. In this study, by adding different amount of HA, inhibitory effect of Cu^2+^ had been eliminated except at the condition of 80 mg L^−1^ Cu^2+^ + 3 g L^−1^ HA.

To better understand the adsorption of Cu^2+^ on HA, the sorption experiments were conducted. The result showed that HA had good sorption capacity for Cu^2+^ at pH = 7.0 and the adsorption data could be well described by Langmuir equation (Figure 1). Based on *q*_m_, and *k*_L_ in Table 1, the equilibrium Cu^2+^ concentrations (*C*_*e*_) at initial Cu^2+^ concentrations of 5–80 mg L^−1^ were calculated and listed in Table 3. Low *C*_*e*_, ranging from 0.258 to 5.19, were obtained except at the exact condition of 80 mg L^−1^ Cu^2+^ + 3 g L^−1^ HA, where *P. aeruginosa* cell with irregular shapes were observed. This is a reasonable explanation for the inhibitory effect at the condition of 80 mg L^−1^ Cu^2+^ + 3 g L^−1^ HA.

**Table 3:** Equilibrium Cu^2+^ concentrations (*C*_e_) at different initial Cu^2+^ concentrations on humic acid (HA)

Initial Cu^2+^ concentrations	3 g L^−1^ HA	6 g L^−1^ HA
5	0.496	0.258
20	2.15	1.08
40	2.80	2.28
80	12.4	5.19

Initial Cu^2+^ concentrations (mg L^−1^); Equilibrium Cu^2+^ concentrations, *C*_e_, (mg L^−1^).

Carboxyl group (–COOH) was the predominant functional groups of HA responsible for Cu^2+^ sorption through both surface complexation and ion exchange (Yang et al., 2015). Surface complexation occurs due to the chemical coordination between –COOH and Cu^2+^, which is an irreversible process mainly with chemical nature. While ion exchange occurs due to the electrostatic attraction between –COO^−^ and Cu^2+^, which is a reversible process mainly with electrostatic nature. Hence, Cu^2+^ needed for the growth of *P. aeruginosa* is always available. Biodegradation was mostly improved at the condition of 40 mg L^−1^ Cu^2+^ + 3 g L^−1^ HA. Interestingly, 6 g L^−1^ HA exhibited no better effect than 3 g L^−1^ HA. This is probably because HA can also absorb onto the bacterial surface (Wightman & Fein, 2001), leading to competitive adsorption between HA and BDE‐209.

Earlier study revealed that the biodegradation pathway of BDE‐209 by degrading bacteria mainly include debromination, hydroxylation, and cleavage of the diphenyl ether bond (Lu et al., 2013; Wang et al., 2016). Herein, in the simultaneous presence of 40 mg L^−1^ Cu^2+^ and 3 g L^−1^ HA, BDE‐209 was degraded in the same mechanism. Debromination is a critical step in the mineralization of BDE‐209 and was improved by adding 40 mg L^−1^ Cu^2+^ and 3 g L^−1^ HA as shown in Figure 4. Hydroxylation reaction is also an important step in degradation of BDE‐209, while the synergistic effect of Cu^2+^ and HA on hydroxylation need further examination.

In summary, a certain amount of soil HA can eliminate the toxic effect of Cu^2+^ on *P. aeruginosa* cell and synergistic effect of Cu^2+^ and HA obviously improve the biodegradation of BDE‐209 by *P. aeruginosa*. To the author's knowledge, this is the first report on the biodegradation of BDE‐209 by *P. aeruginosa* in the simultaneous presence of Cu^2+^ and soil HA. These discoveries may provide substantial support for the potential application of *P. aeruginosa* in bioremediation of e‐waste‐contaminated soils. The future work will focus on applying *P. aeruginosa* to remediate BDE‐209 in e‐waste‐contaminated soils.

## CONFLICT OF INTEREST

The authors declare no conflict of interest.
